# An affordable apparatus for fine‐controlled emulation of buzzing frequencies of bees for the testing hypothesis in buzz interactions

**DOI:** 10.1002/ece3.4290

**Published:** 2018-07-12

**Authors:** Ernani V. Rodrigues, Júlia R. Riguette, Henrique R. C. Pereira, Juliétty A. Tesch, Ary G. Silva

**Affiliations:** ^1^ Graduate School of Education University of São Paulo São Paulo SP Brazil; ^2^ Graduate Program in Ecosystem Ecology University Vila Velha Vila Velha ES Brazil; ^3^ Federal University of ABC São Paulo SP Brazil; ^4^ Group of Solid Mechanics and Structural Impact University of São Paulo São Paulo SP Brazil; ^5^ Instituto Capixaba de Pesquisa Assistência Técnica e Extensão Rural Vitória ES Brazil

**Keywords:** buzz pollination, foraging behavior, pollen harvesting, pollination biology

## Abstract

The buzzing foraging behavior of female bees for pollen harvesting called the attention of early pollination biologists. Flower types that demand this buzzing behavior comprise about 20,000 species of different and phylogenetically unrelated plant taxa, suggesting that it had independently evolved many times among the flowering plants. Between the late 1970s and early 1980s, theoretical papers had modeled the energetics of buzz pollination, but, up to this moment, no hypothesis was experimentally tested concerning the theoretical basis of the energetics of buzz pollination. We present a cost‐effective and simple apparatus, including a digital and highly accurate frequency generator, and a device for the transference of buzz‐frequency energy to the receptive floral unity. The receptive floral unities may comprise the entire or partial androecium, or the tubular corolla, or, in some cases, the whole flower. This apparatus can be easily used in both laboratory and field conditions of research, as natural air currents are avoided, and the response of pollen liberation can be quantitatively measured by pollen grain counts that can be captured by adhesion in slide poured with an isosmotic lactate–glycerol media. The maximum displacement of the hardwire beam/claw system was 0.1170 ± 0.0006 mm @ 150 Hz; 0.021 ± 0.003 mm @ 250 Hz; 0.010 ± 0.001 mm @ 350 Hz; 0.0058 ± 0.0001 mm @ 450 Hz; and 0.0082 ± 0.0005 mm @ 550 Hz. Hypothesis contrasting frequency emission and pollen liberation measured as pollen grain counts may be tested in a species flower type by simple linear regression if pollen counts are normally distributed, or ordinal logistic regression, with non‐normal counts. The comparison among different flower‐type requirements can be tested through appropriate statistical methods for both normally and non‐normally distributed pollen grain counts.

## 1 INTRODUCTION

When we evaluate the pollination process under the perspective of palynology that includes spores, prepollen, and pollen in its scope (De Vernal, 2015; Erdtman, 1963), insect and plant interactions involving pollination had arisen previously to the adaptive radiation of the angiosperms on the Mid‐Cretaceous (Labandeira, Kvaček, & Mostovski, 2007). In fact, it can be located in the Upper Carboniferous, concerning fossil records of *Monoletes*‐type prepollen from a Pteridospermatophyta on the leg of a myriapod *Arthropleura* specimen (Scott & Taylor, 1983), what may suggest spore dispersal or even pollinivory (Labandeira, 1998).

Pollen consumption was definitively demonstrated by fossil records of gut contents and coprolites of arthropods. These examples demonstrate that spores, prepollen, and pollen were important dietary components for Paleozoic insects, suggesting that pollinivory probably was an important precursor to suggested Paleozoic pollination mutualisms (Labandeira, 1997) that occurred between insects and Pteridospermatophyta (Taylor & Taylor, 1993) and Mesozoic gymnosperms (Labandeira et al., 2007).

The ancient pollination services were strongly associated with the consumption of plant tissues, even after the adaptive radiation of the angiosperms, for instance, as in beetle‐pollinated angiosperms (Bernhardt, 2000). The herbivores started their diversification in florivores (McCall & Irwin, 2006) and pollinators (Labandeira, 2000), and in this scenery, some trends had arisen some shifts from the consumption of floral parts, including pollen and ovules, to some other nutritional rewards (Nicholls & Hempel de Ibarra, 2017), such as nectar, since the Late‐Cretaceous (Grimaldi, 1999).

In thermodynamic terms, pollen has a high caloric nutritional cost for plants, as it contains highly energetic metabolites, such as fatty acids, vitamins, proteins, and nucleic acids (Simpson & Neff, 1983). In this context, some constraints in the assessment of pollen had also evolved in the flowering plants, even in specialized bee‐pollinated flowers (Westerkamp, 1997). The poricidal anthers are one of the floral traits that enhance the pollen protection and reduce its loss as they can direct pollen grains to the body of the pollinator. This morphological feature also restricts the spectrum of floral visitors, as, in principle, only a few groups of bees are capable of vibrating anthers and promote buzz pollination (Endress, 1996).

The vast majority of flowers with poricidal anthers are visited by bees that are capable of producing vibrations triggered by contraction of the indirect muscles of flight in their chest. These bees transmit the vibration through their legs to the androecium or even the whole flower, causing pollen release. This process is called buzz pollination or pollination by vibration (Buchmann, 1983) that usually occurs in flowers with poricidal anthers and presents pollen as the only floral reward (Vogel, 1978).

This kind of pollination has a widespread distribution among the angiosperms (Buchmann, 1983), even in plant species which flowers do not have poricidal anthers (Buchmann, 1985), and it is the result of a long history of coevolution and plant–pollinator adaptation (Proença, 1992). While poricidal anther had evolved in independent phylogenetically angiosperm groups, the vibrate habit also appears in phylogenetically distinct groups of bees, showing that it has the characteristics of ecological convergence, which is however a specialized pollination process evolutionarily (De Luca & Vallejo‐Marín, 2013).

The buzzing foraging behavior of female bees for pollen harvesting called the attention of early pollination biologists. Flower types that demand this buzzing behavior comprise about 20,000 species of different and phylogenetically unrelated plant taxa, suggesting that it had independently evolved many times among the flowering plants (De Luca & Vallejo‐Marín, 2013).

Despite the importance of buzz pollination to the natural and agricultural systems, it has received limited attention in the last 30 years (Buchmann, 1983; De Luca & Vallejo‐Marín, 2013). From the late 1970s up to the early 1980s, theoretical papers had modeled the energetics of buzz pollination (Buchmann & Hurley, 1978). However, there are not a consistent number of hypothesis that was experimentally tested, concerning the theoretical basis of the energetics of buzz pollination (Waser, Chittka, Price, Williams, & Ollerton, 1996).

Moreover, so, we present a cost‐effective and simple apparatus, including a digital and highly accurate frequency generator, and a device for the transference of buzz‐frequency energy to the receptive floral unity. The receptive floral unities may comprise the entire or partial androecium, or the tubular corolla, or, in some cases, the whole flower.

## 2 CRAFTING THE APPARATUS

**Figure 1:**
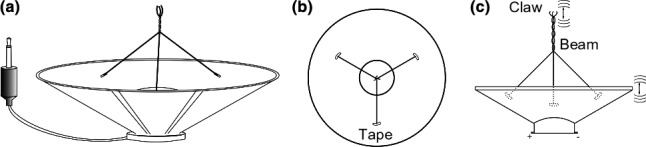
Connector and speaker (a); beam attached to the speakers’ cone using tape (b); hardwire braid and claw (c)

The apparatus (Figure 1) is a regular small speaker with a mono plug inserted in an MP3 player device. A hardwire beam is attached to the speaker's cone, transferring vibration to the flower. The list of parts consisted of (a) a mono P2 plug TS 3.5 mm; (b) a 3″ speaker, 8 Ω; (c) a 6′ long two‐way signal cable, 0.5 mm^2^; (d) an steel hard wire, for the beam and the claw; (e) insulating tape; and (f) soldering iron and solder wire.

The cable is soldered to the TS plug and the speaker pole by pole; then, three hardwire pieces are attached to the speaker's cone with insulating tape in a 120° separation and braid as a central beam, as shown in Figure 1.

The hardwire used for the beam/claw was made from galvanized steel with diameter 1.24 mm, linear density 9 g/m, and maximum tensile strength at 55 kgf/mm^2^.

**Figure 2:**
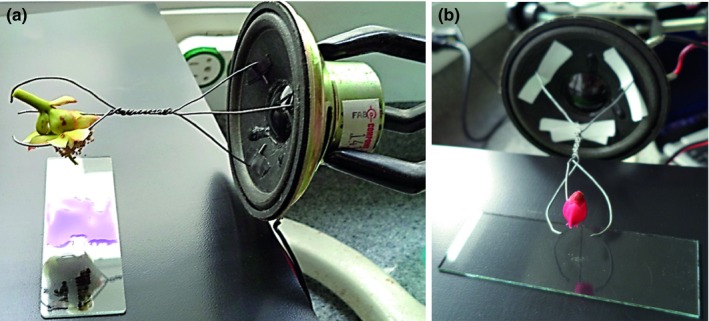
Apparatus in laboratory operation. (a) *Bonnetia stricta* (Nees) Nees ex, Mart. (b) *Gaylussacia brasiliensis* (Spr.) Meissner

At the beam tip, the three hardwire pieces are bent in a claw form to connect the flower's peduncle (Figure 2).

## 3 GENERATING BUZZ

**Figure 3:**
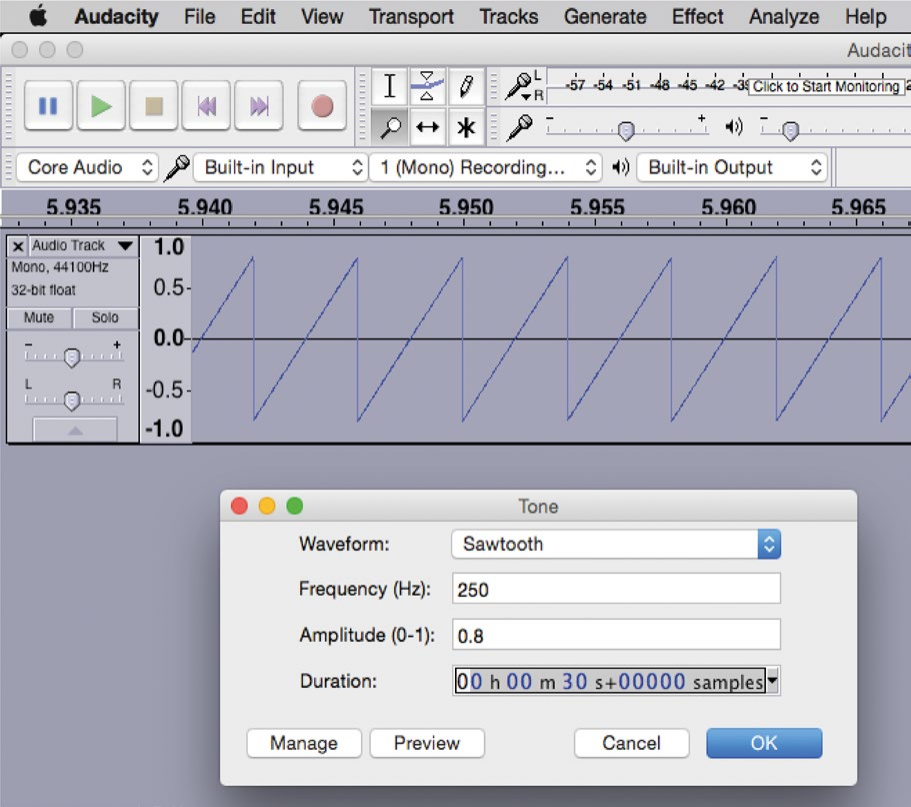
Audacity tone generator screen during the generation of audio files at the frequency of 250 Hz

We used the free multitrack audio editor and recorder Audacity (Figure 3), available at http://audacity.sourceforge.net/. The waveform we used in this experiment was synthesized in the *wave generator* function: Generate > Tone.

We tested several waveforms such as sine, square, and sawtooth in order to emulate the buzz in a controlled frequency range to verify the interaction between the bee's buzz and the flower structure. The sawtooth waveform was taken because of its similarity to the bee's buzz waveform and transient (Esch & Wilson, 1967). Then, for each waveform MP3 files were generated from 150 to 550 Hz with every 25‐Hz interval, and each file was 3 s long. The uncertainty for the frequency value can be measured generating two waveforms with a frequency slightly different and comparing the cycle difference. For 1,000 Hz, in a 1‐min‐long track, the uncertainty is less than ±0.001 Hz, and so we assumed ±0.001 Hz as the uncertainty in frequency value. We kept those files on a laptop where we connected the plug to the phone jack.

## 4 MEASURING THE CLAW DISPLACEMENT

**Figure 4:**
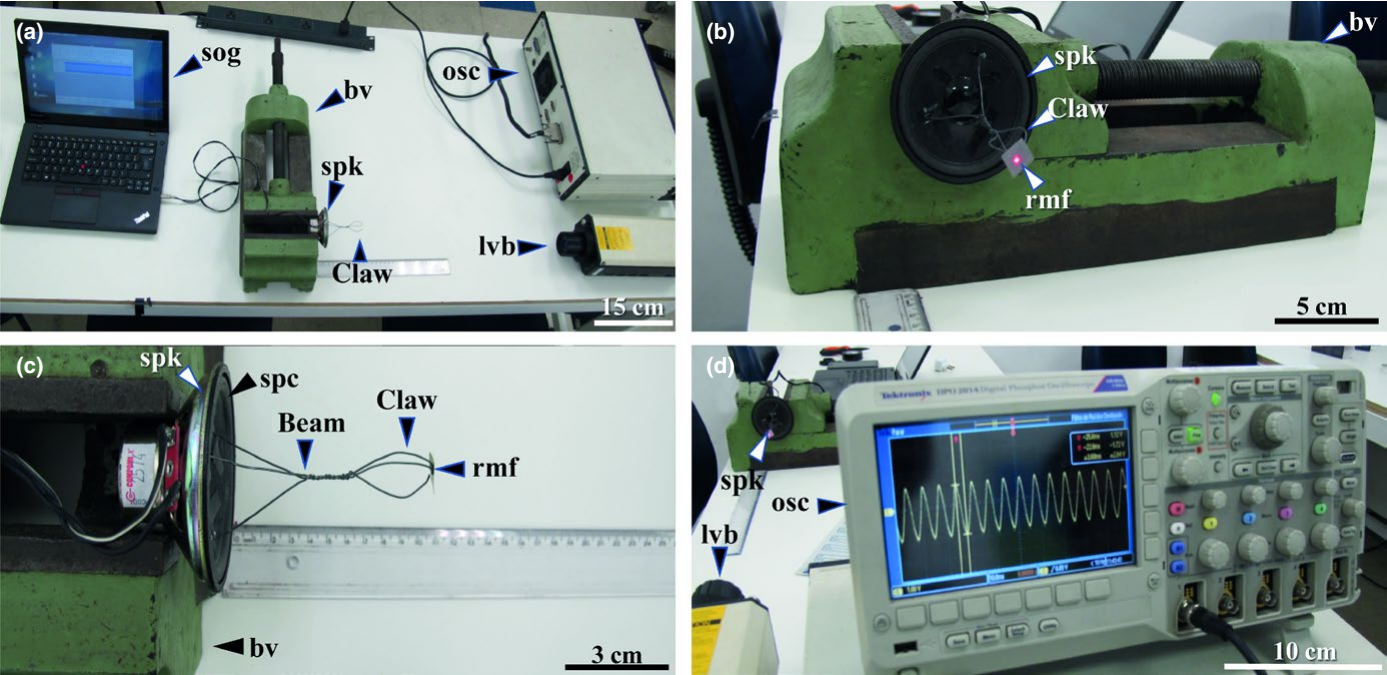
System to measure claw's displacement. (a) Signal output generator (sog), bench vise (bv) fixing the speaker (spk) where the hardwire beam/claw is attached to, the Polytec 3000^®^ LASER vibrometer (lvb), and Tektronix DPO 2014^®^ 100MHz oscilloscope (osc); (b) detail of the speaker (spk) fixed to the bench vise (bv), bearing a metallic reflexive foil (mrf) at the tip of the claw; (c) equatorial view of the speaker (spk) fixed to a bench vise, with the hardwire beam/claw attached to the speaker cone (sc), and the metallic reflexive foil (mrf) at the tip of the claw; (d) laser vibrometer (lvb), in front of the speaker (spk), and oscilloscope (osc) in detail

The maximum displacement of the claw is highly influenced by the amplifier power handed, and the frequency of the wave is generated. Also, the structural design of the speaker and the claw can resonate in certain frequencies, which may change the claw displacement. To verify the displacements amplitudes, we used five main frequencies from 150 to 550 Hz in a 100 Hz steps, covering the working range. We used a system (Figure 4) in which the output signal was taken from a regular 3.5 mm phone jack of a Lenovo T450^®^ laptop, at the maximum volume, and the sawtooth wave amplitude generated in Audacity was placed at 0.8. The beam/claw system weighted 1.08785 ± 0.00001 g and was attached to the cone of the speaker, which was fixed to a bench vise. A metallic reflexive foil weighting 0.02343 ± 0.00001 g was attached to the claw, and a Polytec 3000^®^ LASER vibrometer system was used for measuring the amplitudes of displacement. The vibrometer data were read in a Tektronix DPO 2014^®^ 100 MHz oscilloscopes.

**Figure 5:**
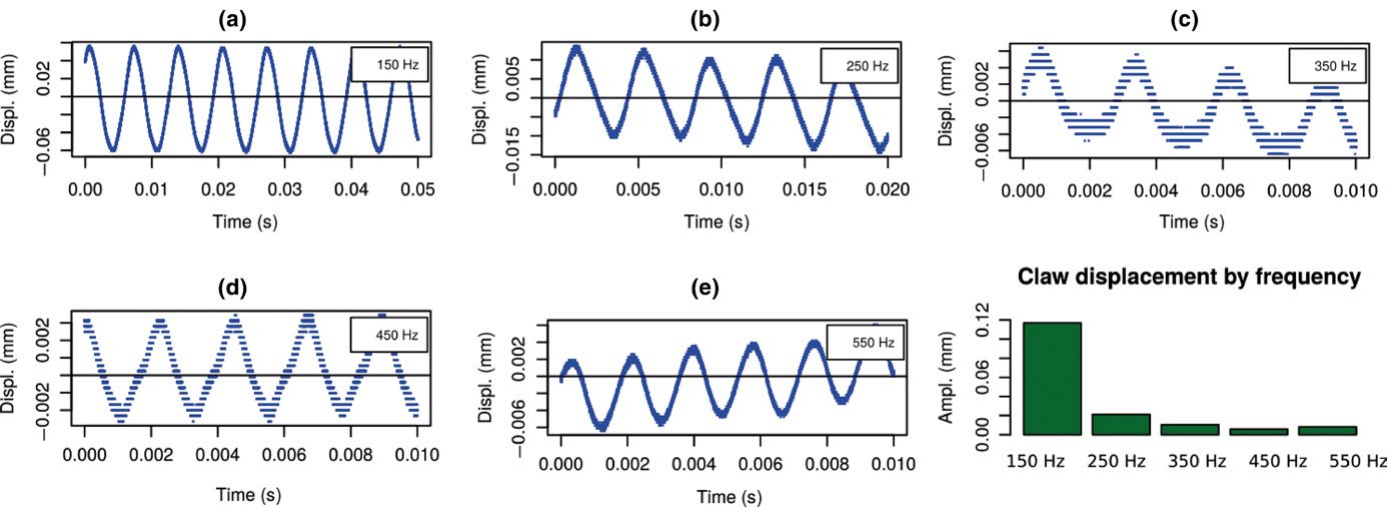
Displacement oscillation (from a to e) and amplitudes measured by the frequency with the laser vibrometer system

The data analysis taken from the oscilloscope is measured in Volts, and the vibrometer factor (40 μm/V) was multiplied by transforming the voltage reading in “mm” of displacement. Then, a peak analysis was processed in R software. The device's claw vibrations are shown in Figure 5 ahead.

The maximum displacement of the hardwire beam/claw system was 0.1170 ± 0.0006 mm @ 150 Hz; 0.021 ± 0.003 mm @ 250 Hz; 0.010 ± 0.001 mm @ 350 Hz; 0.0058 ± 0.0001 mm @ 450 Hz; and 0.0082 ± 0.0005 mm @ 55 0 Hz.

## 5 BUZZ STRESSING AND QUANTITATIVE RESPONSE

The device is operated indoors, in a laboratory free of air currents. The speaker was held on a stand, and the claw as attached to some part of the flower that could primarily receive vibration from the speaker's cone that was transferred to the beam and then to the flower (Figure 5).

Pollen grain of flowers with poricidal anthers is not usually released merely by gravidity, even when they are inverted toward the ground (Buchmann, 1983). Therefore, in each frequency, the sampled flower was upside‐down over a slide (Figure 5) containing five drops of an isosmotic media to avoid the rupture of the pollen grains, composed of a mixture of malachite green and acid fuchsin, and dissolved in lactic acid, glycerol, and water (Alexander, 1980). After a 3‐s‐long vibrate stressing on a sampled flower, the pollen grains that had fallen into the isosmotic media in the slide received a coverslip, and then, they were counted in a light microscope.

Hypothesis contrasting frequency emission and pollen liberation measured as pollen grain counts may be tested in a species flower type by simple linear regression if pollen counts are normally distributed, or ordinal logistic regression, with non‐normal counts. The comparison among different flower‐type requirements can be tested through appropriate statistical methods for both normally and non‐normally distributed pollen grain counts, if necessary, with an eventual scale correction can be made using the ranging standardization (Gower, 1971; Hosmer & Lemeshow, 2000; Zar, 2010).

## 6 CONCLUSION

We present a cost‐effective and simple apparatus, including a digital and highly accurate frequency generator, and a device for the transference of buzz‐frequency energy to the receptive floral unity. The receptive floral unities may comprise the entire or partial androecium, or the tubular corolla, or, in some cases, the whole flower. This apparatus can be easily used in both laboratory and field conditions of research, as air currents could be avoided, and allows hypothesis contrasting frequency emission and pollen liberation measured as pollen grain counts and the comparison among different flower‐type requirements.

## CONFLICT OF INTEREST

None declared.

## AUTHORS’ CONTRIBUTIONS

Ernani V. Rodrigues has designed and made the device and produced the vibratory frequencies files to be used in laboratory. Julia R. Riguete has run the laboratory experiments with the flowers, registering. Henrique R.C. Pereira has designed and carried on the measures in laser vibrometer/oscilloscope system. Juliétty A. Tesch has processed and analyzed the data of the displacement for hardwire beam/claw system. Ary Gomes da Silva has designed the hypothesis and all the experimental approach, developed the technique for collecting pollen grains, and adapted the technique to prevent them from osmotic damages. All the five authors have drafted and revised the final manuscript.
